# Television viewing in Thai infants and toddlers: impacts to language development and parental perceptions

**DOI:** 10.1186/1471-2431-9-34

**Published:** 2009-05-22

**Authors:** Nichara Ruangdaraganon, Jariya Chuthapisith, Ladda Mo-suwan, Suntree Kriweradechachai, Umaporn Udomsubpayakul, Chanpen Choprapawon

**Affiliations:** 1 Department of Paediatrics, Faculty of Medicine Ramathibodi Hospital, Mahidol University, Bangkok, Thailand; 2 School of Community Health Sciences, University of Nottingham, Nottingham, UK; 3 Department of Paediatrics, Prince of Songkla University, Songkhla, Thailand; 4 Department of Research Centre, Faculty of Medicine Ramathibodi Hospital, Mahidol University, Bangkok, Thailand; 5 Health System Research Institute, Ministry of Public Health, Nontaburi, Thailand

## Abstract

**Background:**

Effects of television to language development in infants and toddlers, especially in the Asian children, are inconclusive. This study aimed to (a) study time spent on television in Thai infants and toddlers (age < 2 years), (b) investigate the association between time spent on television (as recommended by the American Academy of Paediatrics (AAP), < 2 hours per day) and language development in Thai 2-year-old children, and (c) explore parental perceptions on television toward their child's development.

**Methods:**

Two hundred and sixty children and their parents were recruited into the study. Time spent on television and parental perceptions on television viewing toward their child's development were recorded during face-to-face and telephone interviews. Language development was assessed at the age of 2 years using the Clinical Linguistic Auditory Milestone Scale (CLAMS), and parents' report. Association between delayed language development and time spent on television viewing, as well as other various parameters such as gender, maternal education and family income, were analysed using a multivariate logistic regression model.

**Results:**

Most Thai infants and toddlers watched television at the age of 6 months, 1 year and 2 years old (98.0, 95.3 and 96.7%, respectively). On average, 1-year-old children watched television 1.23 ± 1.42 hours per day. This increased to 1.69 ± 1.56 hours per day when they were 2 years old. However, watching television longer than 2 hours per day did not associate with delayed language development. On multivariate logistic regression analysis, gender (male) was the only significant factor associated with delayed language development (OR = 6.9, 95% CI = 1.5–31.3). Moreover, 75%, 71%, and 66% of Thai parents believed that television viewing yielded benefits to children's developments.

**Conclusion:**

Thai children commenced watching television at an early age and the amount of television viewing time increased by age. Most parents had positive perceptions to television viewing. The study found no association between time spent on television viewing (≥ 2 hours per day) and delayed language development at the age of 2 years.

Gender (male) was the only variable associated with delayed language development.

## Background

Television has become part of children's daily life. However, effect of television viewing to children and adolescents; in terms of health, social interaction, behaviour, attention and development, is inconclusive [1-5]. Television viewing may provoke either positive or negative impact to developments of children [6]. According to the American Academy of Paediatrics (AAP), electronic screens viewing time should not exceed 2 hours per day [7]. These recommendations are based on the assumption that children who spent their time on screen media may spend less time with their parents; and, thus, may be detrimental to their development [8].

On average, school-aged American children spent 2–5 hours per day watching television [9,10]. However, data regarding exposure to television in very young children (especially in infants and toddlers who were younger than 2 years) is limited, in particular data from the Asian population. A study from the USA demonstrated that 17% of 0- to 11-month-olds, 48% of 12- to 23-month-olds, and 41% of 24- to 35-month-olds watched television more than 2 hours per day [11]. Also, a longitudinal study in the USA reported that children aged 1 1/2 years and 3 1/2 years spent average of 2.2 and 3.6 hours per day, respectively, watching television [12]. Those studies showed that the AAP recommendations have not been obeyed in the real world [13].

Although, there are a number of studies demonstrated the impact of television viewing to school-age children's development, data in infants and toddlers is limited [6]. Previous published data reported inconclusive effect of television viewing to language development in young children, depending mainly on types of television programmes [6]. Some children's television programmes, where onscreen characters speak directly to the child, actively participate, label objects, contain vocabulary words and their definitions, and provide the child to respond, such as Dora the Explorer, Blue's Clues, Dragon Tales, Arthur and Clifford, may enhance children's language development. On the other hand, some programmes that have loose narrative structure and poor language models such as Sesame Street and Teletubbies were associated with reduced vocabulary in the children [14].

Time spent on television, types of programmes and parental control over child's television viewing may be cultural different between the Asian and the western countries. Recent case-control study in Thailand found the negative relationship between television viewing onset, television viewing time, and language development [15]. However, there is no prospective longitudinal research regarding the association between television viewing and language development and parental perception on television viewing in infants and toddlers in Asian countries. This study was carried out with the aims (a) to study the amount of time spent on television viewing in 6-month-, 1- and 2-year-old Thai children; (b) to investigate whether longer time spent on television viewing (≥ 2 hours per day) were associated with delayed language development at the age of 2 years; and (c) to explore parental perceptions on television viewing toward their child's development.

## Methods

### Participants and procedures

The study was a part of the Prospective Cohort Study of Thai Children (PCTC), a community-based longitudinal birth cohort study following up children from birth to 24 years of age. Children who were born during October 2000 and September 2002 at two university-based hospitals (Ramathibodi and Rajavithee Hospitals) in Bangkok, Thailand and their parents were recruited into the study. The study was ethically approved by the National Ethics Committee, the Ministry of Public Health of Thailand, and the Ethics Committees of both hospitals.

Two hundred and sixty children and their parents agreed to participate into the study and signed inform consents. Data regarding amount of time spent on television viewing were collected during face-to-face interview with the parents when the children were 6 months, 1 and 2 years old. The parental perceptions on television viewing were recorded during the telephone interviews. Language development was evaluated when all children were 2 years old, by qualified developmental and behavioural paediatricians. Of the 260 children and parents recruited into the study; 260, 256 and 203 children remained in the study after they were 6 months, 1 and 2 years old; only 220 parents completed the telephone interview. The remaining had either withdrawn from the study because of their inconvenience or loss of contact.

### Measurements

#### Television viewing

At the age of 6 months, parents were asked to answer the question "How often did your child was placed in front of the television?" and were asked to classify (their child's television exposure) as "Never" (not at all), "Sometimes" (less than 4 days per week) and "Often" (4 days or more per week). The amount of time spent each day on watching television was not recorded at the age of 6 months. At the age of 1 and 2 years, the parents were asked to answer the question "How many minutes/hours has your child spent on watching television a day?". The amount of time spent on television viewing was then recorded. However, names and types of the television programmes were not documented.

#### Assessment of language development

Assessment of delayed language development was carried out by using standardized instrument, modified Clinical Linguistic Auditory Milestone Scale (CLAMS) [16]. The CLAMS was modified to fit Thai cultures and was translated from English into Thai. All pictures in modified CLAMS depicted the same items as the original CLAMS, except the picture of a dog. The dog picture was administered to children in all regions of the country except in the south, where dog is not a common pet. Therefore, a goat, which is usually seen in most families, was selected to substitute the dog picture. All administrators using the modified CLAMS received training by the developmental and behavioural paediatrician and were supervised during its administration. "Delayed language development" was documented when the child failed all items at the 21 months of age which implied the child was functioning at less than 87.5% age expectations.

#### Parental perceptions on television viewing

Parental perceptions on television viewing were assessed in three main areas of child development: attitude toward cognitive development, attitude toward language development and attitude toward social development. Parents were asked to answer the question: "Do you think watching television would likely enhance or delay or would not affect your child's cognitive/language/social development?". Responses were categorised as positive (television enhances child's development), negative (television delays child's development) and no effect (television has no effect on child's development).

### Analyses

Statistical analyses were carried out with SPSS 11.5 (SPSS, Chicago, IL, USA). Descriptive analyses were used to ascertain the amount of time spent on television viewing and to analyse data on the parental perceptions toward television viewing. Association between delayed language development and time spent on television viewing, as well as other various parameters, were analysed using a multivariate logistic regression model. Odds ratios and 95% confidence intervals were reported.

## Results

The percentages of children who watched television at the age of 6 months, 1 year and 2 years were 98, 95.3 and 96.7, respectively. Of the 260 six-month-old children; 43.5, 54.2 and 2.3% were "often", "sometimes" and "never" watched television, respectively. Moreover, 22.4% of 1-year-old children spent ≥ 2 hours per day on television viewing, and 38.4% of 2-year-old children watched television ≥ 2 hours per day. The average television viewing time for the 1- and 2-year-old children was 1.23 ± 1.42 and 1.69 ± 1.56 hours per day, respectively.

On multivariate analysis, only gender was significantly associated with delayed language development (OR = 6.9, 95% CI = 1.5–31.3). There was no association between delayed language development and other variables (maternal education, monthly family income, number of children in family, number of household televisions, television in child's bedroom and time spent on television viewing) (Table 1).

**Table 1:** Associations between delayed language development at the age of 2 years and various parameters.

**Parameters**	**Language development at 2 years old**		**Odds ratio** **(95% CI)**
		
	**Normal (n = 187)**	**Delayed (n = 16)**	
**Gender**			
**Male**	94	14	6.9 (1.5–31.3)
**Female**	93	2	1.0

**Maternal education**			
**≤ 12 years**	141	12	1.0 (0.3–3.2)
**> 12 years**	46	4	1.0

**Monthly family income (in Thai baht, 1US$ = 40 baht)**			
**≤ 50,000**	175	15	0.4 (0.05–3.7)
**> 50,000**	12	1	1.0

**Number of children in family**			
**≤ 2**	168	15	0.6 (0.07–4.7)
**> 2**	19	1	1.0

**Number of televisions in households**			
**≤ 2**	150	13	0.9 (0.3–3.5)
**> 2**	37	3	1.0

**Television in child's bedroom**			
**Yes**	123	7	0.4 (0.1–1.1)
**No**	64	9	1.0

**Time spent on television viewing at 2 years old**			
≥ 2 hours per day	74	4	0.5 (0.2–1.6)
< 2 hours per day	113	12	1.0

Adjusted odds ratios with 95% confidence intervals were calculated using multivariate logistic regression adjusting for all factors presented in the table.

Of the 203 two-year-old children, 16 (7.9%) had "delayed" language development, and all watched television since they were 6 months old. Also, of the 16 children with delayed language development, 4 spent ≥ 2 hours per day on television viewing, and 12 children spent < 2 hours per day on television. Whilst; of the 187 children who had normal language development, 74 spent ≥ 2 hours per day on television viewing, and 113 children spent < 2 hours per day on television. There was no association between delayed language development and longer time spent on television viewing (≥ 2 hours per day) (OR = 0.5, 95%CI = 0.2–1.6) (Table 1).

**Figure 1:**
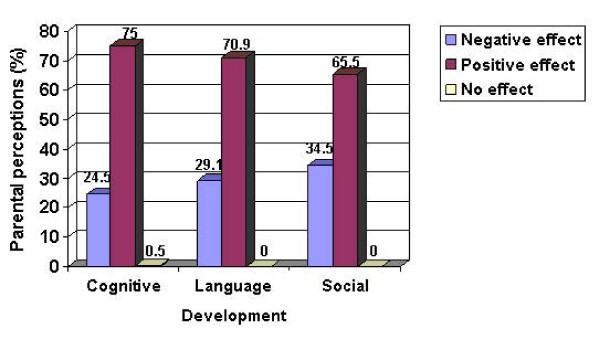
**Parental perceptions on the effects of television viewing toward children's development**. 75, 70.9 and 65.5% of parents believed that television viewing had positive effect to children's cognitive, language and social development.

In addition, 75, 70.9 and 65.5% of parents who completed the interview believed that television viewing could enhance their children's cognitive, language and social development (Figure 1).

## Discussion

This longitudinal birth cohort study presented data on: (a) the amount of time spent on television viewing in infants and toddlers, (b) the association between time spent on television viewing and language development, and (c) parental perceptions on television viewing toward their children's development.

The definition of "watching television" in this (and other) study has to be carefully evaluated. Watching television in the very young child (especially in the child who is younger than 1 year old) is difficult to evaluate. In this study, we assume that children, who were left to expose to television would be able to learn and understand words and pictures on television; thus, would regard as watching television. In addition, with our limited resources, we were able to record broad frequencies of television viewing for 6 months old children. Although, recording television viewing time with viewing diaries is more reliable than parents' report, it requires high parent's effort and cooperation. The amount of viewing time recorded by parents' self report may tend to be underestimated, and is a more-to-common practice.

According to the AAP, exposure to television should be restricted in children who are younger than 2 years old [7]. However, 98.0% of 6-month-old, 95.3% of 1-year-old and 96.5% of 2-year-old children in Thailand watched television. These are comparable with other studies from different countries where more than 90% of children younger than 2 years old were reported to watch television [10,17-19]. Findings from our and other studies reflect that the AAP guidelines have not been heeded.

The increased time spent on television viewing was found when the children were older. Time spent on television viewing increased from 1.23 ± 1.42 hours per day at the age of 1 year to 1.69 ± 1.56 hours per day when the children were 2 years old. A study from the USA also documented the increased time spent on television viewing in children during their first 3 years [11]. However, time spent on television is cultural and socioeconomically dependent, varies amongst families, and is likely to be influenced by habit of parents and other members in the family [20].

One of the intentions of this study was to assess the association between delayed language development and the maximum time spent on television recommended by the AAP [7]. However, only seven two-year-old children followed the AAP guidelines (not watching television). Thus, we have analysed the association between delayed language development and time spent on television viewing at ≥ 2 or < 2 hours per day. No association between delayed language development at the age of 2 years and time spent on television viewing (≥ 2 hours per day) was found. However, the only 16 children with delayed language development detected in the study may not have been adequately powered to identify any association. In contrast, other study proposed that exposure to television before the age of 3 years could deteriorate children's cognitive development, thus, resulted in lower reading performance in early elementary school [21]. Longer follow-up (e.g. at the age of 3 or 4 years) should illustrate clearer direction.

Factors responsible for impaired language development are complex and have not been clearly identified. However, evidence has suggested that gender and genetics may involve in these intricate mechanisms. Delayed language development was observed amongst siblings who had family history of language impairment [22]. Furthermore, other studies proposed that boys are more likely to have impaired language development than girls, possibly due to genetics and neurobiological factors [23-25]. Our study, on the multivariate analysis, has confirmed this observation (OR = 6.9, 95% CI = 1.5–31.3). The study found no association between maternal education and monthly family income and delayed language development, which inconsistent with other studies [25-27]. This is probably due to our sample population was ascertained through two institutions from certain geographical areas of the country (Bangkok). Therefore, this population may not be representative of the Thai population and might affect the association between delayed language development and socioeconomic status.

Findings from this study showed that 64% of 2-year-old children in Thailand had television set in their bedrooms. According to the AAP recommendations, this was not a good practice, as television sets should be removed from children's bedrooms [7]. Again, this recommendation may not be simply applied to the environment and the culture in Thailand (and, probably, other Asian countries), where parents would prefer their child staying in the same room with them until the school age. Furthermore, in many low socioeconomic status families in Thailand, only 1–2 rooms are available in their houses; and that all family members would be forced to stay in the same room.

Regarding types of programmes, although evidence has suggested that educational television programmes may increase school readiness in children [5,28], the benefits of these programmes are questionable in infants [14]. A recent study has suggested that only the infant-directed educational programme with parental co-viewing, not the general educational programme for children, could enhance infant-mother interactions [17]. This type of program with parent co-viewing may improve children's long-term developmental outcome [29]. Meanwhile, adult programmes could provide negative effects to cognitive and language development [30]. Our study did not record types of television programmes watched by the children.

The positive parental perceptions on television viewing toward children's development were demonstrated for the first time in Thailand. Other study in the USA has previously documented similar findings [19]. Parents believed that screening media (e.g. television, DVD, video), if appropriately used, are educational and useful to their child's brain development [19]. Those positive parental attitudes on television viewing may be influenced by television programmes, which have claimed to have educational value for children. However, positive parental perceptions may be deleterious to children, if parents do not carefully concern about the types of programme. A number of research demonstrated detrimental impact of the television viewing to cognitive and social development, if the content in the programmes was not appropriated to children [2,12]. Therefore, parents should closely monitor contents of the programmes and should decide appropriately which television programmes are useful for their child.

In this study, modified CLAMS was used to identify children with delayed language development. Different cultures may also limit the use of original CLAMS in identifying children with delayed language development. We have also concerned that administering only the items at 21 months of age may affect a number of children with delayed language development reported in this study.

## Conclusion

This is the first prospective longitudinal report in Thailand where the amount of time spent on television viewing, as well as the association between television viewing and language development and parental perceptions on television viewing, were studied. In Thailand, most children (> 95%) exposed to television at the very young age (6 months or less). However, this did not associate with delayed language development at the age of 2 years. Gender (male) was the only significant factor associated with delayed language development (OR = 6.9, 95% CI = 1.5–31.3). Parents in Thailand expressed their positive perceptions in the value of television viewing to their child's development. Appropriate information based on our findings should be disseminated to paediatricians and parents in order to help them understand the risks and benefits of television viewing.

## Competing interests

The authors declare that they have no competing interests.

## Authors' contributions

NR conceived and designed the study, supervised all the work related to the study and edited the manuscript. JC collected, analysed the data, wrote, and edited the manuscript. LM, UU and CC involved in designing, analyzing data and supervised the study. SK collected and analysed data and involved in drafting manuscript. All authors read and approved the final manuscript.

## Pre-publication history

The pre-publication history for this paper can be accessed here:


